# Molecular subtype conversion in CTCs as indicator of treatment adequacy associated with metastasis-free survival in breast cancer

**DOI:** 10.1038/s41598-022-25609-0

**Published:** 2022-12-05

**Authors:** E. S. Grigoryeva, L. A.Tashireva, V. V. Alifanov, O. E. Savelieva, S. V. Vtorushin, M. V. Zavyalova, O. D. Bragina, E. Y. Garbukov, N. V. Cherdyntseva, E. L. Choinzonov, V. M. Perelmuter

**Affiliations:** 1 The Laboratory of Molecular Oncology and Immunology, Cancer Research Institute, Tomsk National Research Medical Center, Russian Academy of Sciences, Tomsk, Russia; 2 The Department of General and Molecular Pathology, Cancer Research Institute, Tomsk National Research Medical Center, Russian Academy of Sciences, Tomsk, Russia; 3 The Department of Nuclear Medicine, Cancer Research Institute, Tomsk National Research Medical Center, Russian Academy of Sciences, Tomsk, Russia; 4 The Department of General Oncology, Cancer Research Institute, Tomsk National Research Medical Center, Russian Academy of Sciences, Tomsk, Russia; 5 The Department of Head and Neck Surgery, Cancer Research Institute, Tomsk National Research Medical Center, Russian Academy of Sciences, Tomsk, Russia

**Keywords:** Breast cancer, Metastasis, Chemotherapy

## Abstract

Molecular subtype of breast cancer has a great clinical significance and used as one of the major criteria for therapeutic strategy. Recently, for anticancer therapy, the trend for oncologists is the predominant determination of biomarkers in the existing foci of the disease. In the case of adjuvant therapy prescribed for distant metastases prevention, CTCs could be a suitable object for investigation. CTCs as one of the factors responsible for tumor metastatic potential could be more convenient and informative for evaluation of hormone receptors, Ki-67 and HER2 expression, which are determine molecular subtype in breast cancer patient. In our study, we aimed to investigate the molecular subtype discordance between the primary tumor and CTCs in breast cancer patients. We established conversion of molecular subtype in most of the cases. Namely, conversion was detected in 90% of untreated patients and in 82% of breast cancer patients treated by neoadjuvant chemotherapy. At the same time, molecular subtype conversions in patients treated by neoadjuvant chemotherapy were more diverse. Molecular subtype conversions resulted more often in the unfavorable variants in circulating tumor cells. We stratified all patients according to the adequacy of treatment against converted CTCs molecular subtype. Our study revealed that good response to neoadjuvant chemotherapy observed in case of adequate therapy, namely, when chemotherapy scheme was sufficient against CTCs. It turned out that patients with inadequate therapy were characterized by decreased simulated 5-year metastasis-free survival compared to patients who received appropriate therapy. Thus, detection of molecular subtype conversion in circulating tumor cells could be a perspective tool for optimization of antitumor therapy.

## Introduction

Breast cancer patients often experience a lengthy disease process that can extend for years. During this period, it could be necessary to have a correction of treatment if progression occurs. A growing body of evidence suggests that cancer cells can change surface molecular signature during tumor progression. Molecular subtype of primary tumor has a great clinical significance and used as one of the major criteria for therapeutic strategy. Discordance of hormone receptor (HR) and HER2 overexpression and, consequently, molecular subtype conversion in disseminated tumor cells could be dramatic. Almost all the studies we found were devoted to comparing of HR and HER2 status in primary lesion and metastatic sites^1–3^. Wherein, the frequency of HR/HER2 status discordance varies widely from 10 to 56% for ERα, 25 to 49% for PR and 3 to 16% for HER2^4–6^. Given the HR/HER2 status discordance such relevant sources as ESMO, ASCO and NCCN clinical guidelines recommend re-biopsy of metastatic lesion to confirm presence of target molecules in tumor cells^7–9^. Often, conventional re-biopsies of distant metastatic sites are not feasible or highly invasive. Therefore, alternative methods are required for HR/HER2 status confirmation, including liquid biopsies.

Circulating tumor cells (CTCs) have been validated as a prognostic factor of poor overall survival in both early and metastatic breast cancer^10,11^. Thus, CTCs as one of the factors responsible for tumor metastatic potential could be more convenient and informative for determination of surface molecules alterations during tumor progression.

The purpose of our study is not to evaluate the discordance in HR expression and HER2 status between the primary tumor and CTCs, but to establish discordance of molecular subtype in CTCs compared to the primary lesion. Hereafter, we will use the term “conversion” to refer to this phenomenon. The term “conversion” may not be very eligible, as it generally characterizes the transformation of one state to another and, perhaps, we could not be sure to dealing with true conversion in all cases. One cannot say whether the cellular interface changes during invasion and intravasation, or whether minor subpopulations in subsets of tumor cells are more prone to migrate and become CTCs. Apparently, both mechanisms could be involved in different proportions. CTCs comprise a subset of phenotypically different tumor cells with diversity of expressional characteristics, including HR and HER2. In our study, we established molecular subtype in total pool of CTCs in a similar way that pathologists determine the molecular subtype of the primary tumor. We deliberately use the term “conversion” of molecular subtype, taking into account, its incomplete relevance to the context, due to the convenience of interpreting the results obtained on the discrepancy between the molecular subtype of the primary tumor and the total pool of CTCs.

For clinical practice, the phenomenon of molecular subtype conversion in CTCs could be crucial for selecting the appropriate treatment scheme. Compared to luminal breast cancer, triple-negative and HER2+ breast carcinomas commonly have strong invasiveness, shortened survival and a two–threefold increase in the tumor relapse rate^12^. Therefore, conversion of CTC molecular subtype to these subtypes could be considered as unfavorable conversion variants. We suppose that considering the modification of the CTC molecular subtype, it will be possible to change the chemotherapy regimen and more effectively act on the disseminated tumor cells that can initiate metastasis. This approach might be a promising method of preventing breast cancer metastatic disease.

## Materials and methods

### Cell culture and antibody testing

We used MCF-7 (luminal A subtype) and BT-474 (HER2+) cell lines of human breast adenocarcinoma for antibody testing. Cells were maintained in Dulbecco’s Modified Eagle’s Medium (DMEM) supplemented with 10% fetal bovine serum, 100 μg/ml streptomycin, 100 U/ml penicillin, and 10 mM HEPES in cell culture flasks at 37 °C under a 5% CO_2_ humidified atmosphere. Cells were treated with trypsin (0.25%) for 10 min to detach them from culture flasks, then washed to remove trypsin-containing medium. Finally, the cell pellet was resuspended in phosphate buffered saline (DPBS, Gibco) and analyzed by flow cytometry. Sample viability was assessed with 7-aminoactinomycin D. Each cell suspension was labeled with monoclonal antibodies: BV570-anti-CD45 (clone HI30, mouse IgG1, Sony Biotechnology, USA), BV650-anti-EpCam (clone 9C4, mouse IgG2b, Sony Biotechnology, USA), AF488-anti-ERα (clone AER314, mouse IgG1, Novus Biologicals, USA), APC-anti-PR (clone PR484, mouse IgG1, Novus Biologicals, USA) and AF750-anti-HER2 (clone 24D2, mouse IgG1, BioLegend, USA). Intracellular (EpCam, ERα and PR) antigens were stained using BD Cytofix/Cytoperm kit (BD Biosciences, USA) according to the manufacturer’s instructions. Immunofluorescence was measured by Novocyte 3000 (ACEA Bioscience, USA), and the data were analyzed using NovoExpress software.

### Patients

The prospective study included 43 patients with invasive breast carcinoma of no special type (IC NST) T_2-4_N_0-3_M_0_, admitted for treatment to Cancer Research Institute, Tomsk National Research Medical Center (TNRMC). Sixteen out of forty-three breast cancer patients were treated by neoadjuvant chemotherapy (NAC). Blood samples from five patients were analyzed before treatment, so NAC did not affect the phenotype of the tumor cells. We pooled this group with untreated patients while analyzing conversion variants and their association with clinicopathological parameters. The remaining 11 breast cancer patients underwent phenotyping after treatment by NAC. Therefore, we evaluated the phenotype of tumor cells in this group separately. In all cases, venous ethylenediaminetetraacetic acid (EDTA) blood samples were taken before surgery.

The study was approved by the local ethics committee of the Tomsk NRMC Institutional Review Board (17 June 2016, the approval No. 8), and informed consent was obtained from all patients prior to analysis. Patients were treated according to ESMO Clinical Practice Guidelines^13^.

### Immunohistochemical analysis

After surgical removal, invasive breast tumor specimens were sent to a pathologist for conducting the diagnostic IHC. Evaluation of ERα, PR, HER2 and Ki-67 expression by IHC was performed using Leica Bond-Max automatic immunostainer (Leica, Bannockburn, IL) with Leica Refine detection kit according to standard automated protocols. Paraffin blocks were cut in a rotary microtome (Leica, Germany) to obtain 4 μm thick slices, which were mounted on poly-l-lysine microscope slides (Thermo Fisher Scientific, USA) and kept at 50 °C for 20 min. Next, tissue sections were deparaffinized and rehydrated according to the Leica Bond protocol. Antigen retrieval for PR antibody was performed with Bond Solution #1 (Leica Biosystems, equivalent to citrate buffer, pH 6.0) and for ERα, HER2, Ki-67 was performed with Bond Solution #2 (Leica Biosystems, equivalent to EDTA buffer, pH 9.0) at 100 °C for 20 min. Primary antibodies ERα (RTU, clone 6F11, mouse, Leica Biosystems, Germany), PR (RTU, clone 16, mouse, Leica Biosystems, Germany), HER2 (1:1000, polyclonal, rabbit, Dako, USA) and Ki-67 (1:250, clone SP6, rabbit, Sigma-Aldrich, USA) was incubated for 25 min at RT. The primary antibody was detected with use of the Bond Polymer Refine Detection kit (Leica Biosystems, Germany), and diaminobenzidine (DAB) chromogen. Counterstaining was performed with hematoxylin. Samples were considered positive for PR and ER if at least 1% of nuclei expressed these receptors, as recommended by the American Joint Committee on Cancer. Samples were Ki-67 positive if 20% of cells expressed Ki-67. HER2 positivity was defined as at least 10% of tumor cells with a strong membrane staining score or a weak/moderate staining score accompanied by a positive fluorescent in situ hybridization result. Breast cancers were divided into four subtypes according to ER/PR, HER2 and Ki-67 expression: luminal A (ER/PR-positive, HER2-negative), luminal B (HER2−) (ER/PR-positive, HER2-negative, Ki-67-positive), luminal B (HER2+) (ER− or PR-positive, HER2-positive), HER2+ (ER/PR-negative, HER2-positive), and triple-negative (ER/PR-negative, HER2-negative) subtypes^14^.

### Fluorescent in situ hybridization

HER2 amplification was assessed by fluorescent in situ hybridization using a KBI-10701 probe (Kreatech, USA). The deparaffinized section was treated in a pretreatment solution at 95 °C for 20 min, followed by protease digestion at RT for 4 min. Next, 20 μL of hybridization solution containing directly labeled probes was added to the slide. Sections allowed to hybridize overnight using the ThermoBrite (Abbott Molecular, USA). After washing, cell nuclei were stained with DAPI (PerkinElmer, USA). Sections were placed under glass slides using Fluorescence Mounting medium (Dako, USA) and examined with a ZEISS Axio Imager 2 (Zeiss, Germany).

### Analysis of HER2 expression in breast cancer cells by flow cytometry

Samples with differential expression of HER2 (score 0, 1, 2, 3) assessed using IHC were selected for further flow cytometry analysis. Frozen tumor fragments were thawed at room temperature, sliced into smaller pieces, and placed in a falcon tube containing 1 mL of DMEM medium supplemented with 5% FBS and homogenized using MediMachine (BD Biosciences, USA). The obtained cell suspension was filtered through 70 μm size-pore cell strainer (Corning Falcon™ Cell Strainer), washed twice in PBS by centrifugation at 400×*g*, 10 min. The single cell suspension was labeled with specific antibodies against CD45, EpCam and HER2 to establish the concordance between HER2 expression evaluated by IHC and immunofluorescence detected by flow cytometry. Sample viability was assessed to 7-aminoactinomycin D. Antibody against pan-leukocyte marker CD45 was used for immune cells discrimination. The staining protocol was similar as extracellular staining of CTCs given below in the section Flow cytometry.

### Blood specimen collection and processing for CTCs immunophenotyping

Blood samples were collected EDTA pre-coated 9 mL tubes, and then incubated at 37 °C for 1.5 h. White blood cells were aspirated from thin white layer between plasma and red blood cells after their sedimentation. The obtained cell concentrate washed in 2 mL Cell Wash buffer (BD Biosciences, USA) by centrifugation at 800×*g* for 15 min.

### Flow cytometry

CTCs detection was performed by two steps using flow cytometry. Surface markers (CD45, EpCam, HER2) were stained by first step, intracellular staining (EpCam, ERα, PR, Ki-67) was performed on second step. Samples were incubated at RT for 10 min with 5 μL of Fc Receptor Blocking Solution (Sony Biotechnology, USA). Next monoclonal antibodies were added and incubated at RT for 20 min: 5 μL BV570-anti-CD45 (clone HI30, mouse IgG1, Sony Biotechnology, USA), BV650-anti-EpCam (clone 9C4, mouse IgG2b, Sony Biotechnology, USA), AF750-anti-HER2 (clone 24D2, mouse IgG1, BioLegend, USA). The unstained control and antibody quality control were performed. The isotype antibodies at the same concentration were added to the isotype control sample. After incubation, erythrocytes were lysed by 250 μL OptiLyse buffer (Beckman Coulter, France) at RT for 10 min in dark and washed in 1 mL Cell Wash buffer (BD Biosciences, USA) at 800 g for 6 min.

For intracellular staining cells were permeabilized by 250 μL BD Cytofix/Cytoperm (BD Biosciences, USA) at 4 °C for 30 min in the dark and washed twice in 1 mL BD Perm/Wash buffer (BD Biosciences, USA) at 800 g for 6 min. After samples were diluted in 50 μL BD Perm/Wash buffer (BD Biosciences, USA) and incubated at 4 °C for 10 min in dark with 5 μL of Fc Receptor Blocking Solution (Human TruStain FcX, Sony Biotechnology, USA). Next monoclonal antibodies were added and incubated at 4 °C for 20 min: 5 μL BV650-anti-EpCam (clone 9C4, mouse IgG2b, Sony Biotechnology, USA), AF488-anti-ERα (clone AER314, mouse IgG1, Novus Biologicals, USA), APC-anti-PR (clone PR484, mouse IgG1, Novus Biologicals, USA), BV421-anti-Ki-67 (clone 16A8, mouse IgG2a, Sony Biotechnology, USA). The appropriate isotype antibodies at the same concentration were added to the isotype control sample. After incubation, samples were washed in 1 mL Cell Wash buffer (BD Biosciences, USA) at 800×*g* for 6 min. After samples were diluted in 100 μL Stain buffer (Sony Biotechnology, USA). MCF‐7 cells were used as positive control and U937 cells were used as negative control. Compensation beads (VersaComp Antibody Capture Bead kit, Beckman Coulter) was used (data not shown). The immunofluorescence was analyzed on the Novocyte 3000 (ACEA Biosciences, USA).

### Statistical analysis

The data was analyzed using the GraphPad Prism 9 (GraphPad Software, San Diego, CA, USA). A comparison of the frequency of conversion variants in molecular subtypes was performed using the two-sided Fisher test. Data simulating tool was used to increase sample size with further survival analysis by the Kaplan–Meier method. Survival was assessed by Cox Proportional Hazard Models. p < 0.05 was considered statistically significant.

## Results

Our study included 43 CTC-positive breast cancer patients. The full clinicopathological parameters of the breast cancer patients are presented in Table 1.

**Table 1:** Clinicopathological characteristics of patients.

Parameter	Frequency, % (n)
**Age**	
< 35	6.98% (3/43)
35–50	30.23% (13/43)
> 50	62.79% (27/43)
**Menopause**	
No	34.88% (15/43)
Yes	65.12% (28/43)
**Tumor size**	
T1	37.21% (16/43)
T2	53.49% (23/43)
T3	4.65% (2/43)
T4	6.98% (3/43)
**Stage**	
IA	32.56% (14/43)
IIA	39.53% (17/43)
IIB	18.60% (8/43)
IIIB	6.98% (3/43)
IIIC	2.33% (1/43)
**Molecular subtype**	
Luminal A	39.53% (17/43)
Luminal B	53.49% (23/43)
Triple-negative	6.98% (3/43)
**Tumor grade**	
1	18.60% (8/43)
2	67.44% (29/43)
3	13.95% (6/43)
**Estrogen receptor α**	
Positive	93.02% (40/43)
Negative	6.98% (3/43)
**Progesterone receptor**	
Positive	79.07% (34/43)
Negative	20.93% (9/43)
**HER2**	
Positive	13.95% (6/43)
Negative	86.05% (37/43)
**Ki-67 expression**	
< 20%	53.49% (23/43)
> 20%	46.51% (20/43)
**Lymph node metastasis**	
Yes	30.23% (13/43)
No	69.77% (30/43)
**Distant metastasis**	
Yes	6.98% (3/43)
No	93.02% (40/43)
**Neoadjuvant chemotherapy**	
Yes	27.91% (12/43)
No	72.01% (31/43)

The mean of CTC number in peripheral blood of breast cancer patients was 80 (9–324) cells/ml. There are no significant differences of CTCs quantity in groups divided according to clinicopathological parameters (data not shown), except molecular subtype of primary tumor. CTCs count in patients with luminal A subtype was significantly higher compared to patients with luminal B (HER2−) or luminal B (HER2+) (p = 0.03). Median of CTCs number in luminal A patients was 125 (46–152) cells/mL, while in luminal B (HER2−) and luminal B (HER2+) patients were 37 (21–74) cells/mL and 42 (20–56) cells/mL, respectively.

### Antibody testing in culture cells

**Figure 1:**
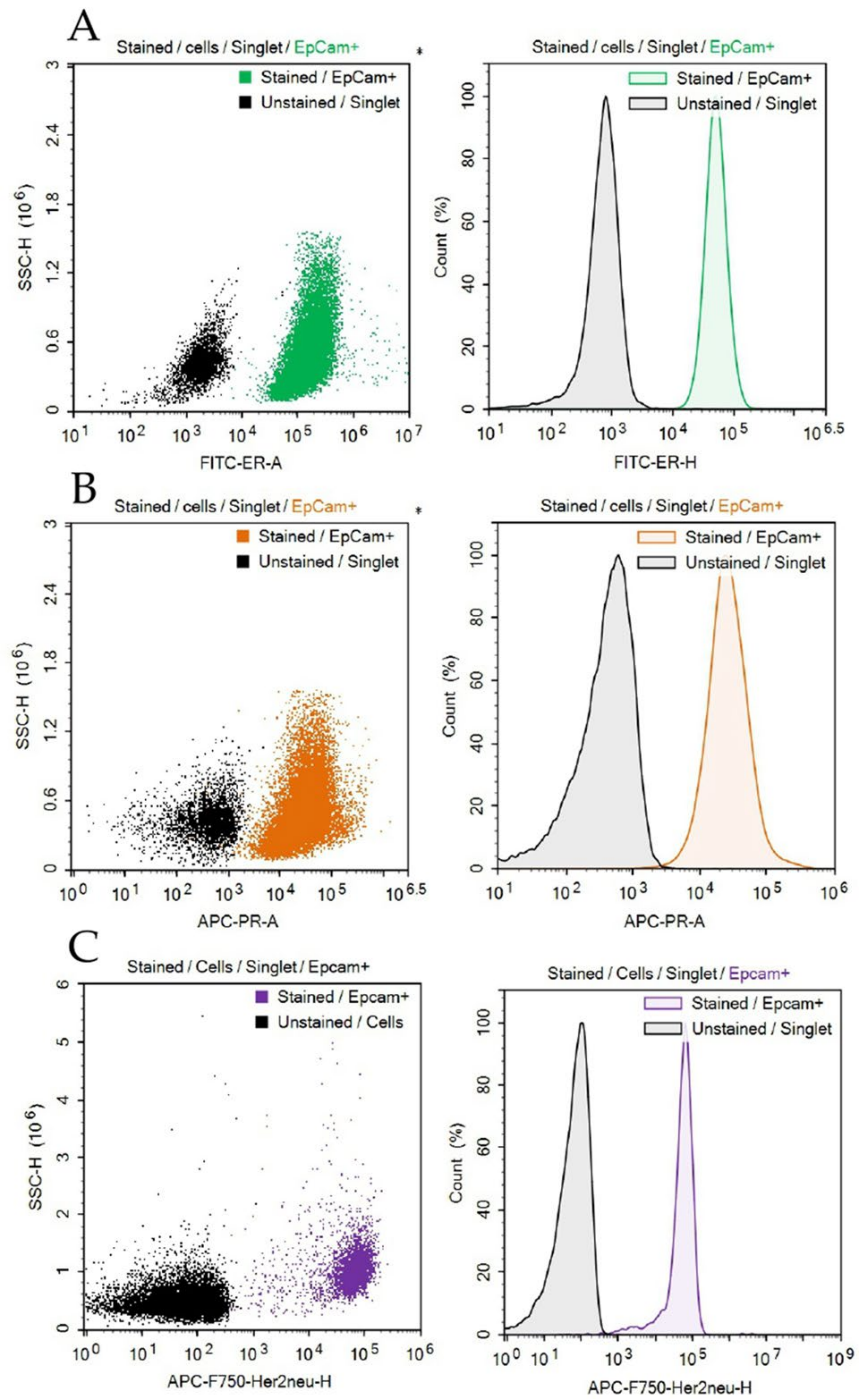
(**A**) Comparison between MCF-7 labeled with anti-ERα antibody and non-labeled MCF-7 cell culture; (**B**) comparison between MCF-7 labeled with anti-PR antibody and non-labeled MCF-7 cell culture; (**C**) comparison between BT-474 labeled with anti-HER2 antibody and non-labeled BT-474 cell culture.

We used MCF-7 (luminal A) and BT-474 (HER2-enriched) cell lines for labeling with ERα, PR and HER2 antibody-fluorophore conjugates to ensure that flow cytometry is relevant to distinguish the molecular subtype of breast cancer. The mean fluorescence intensity (MFI) of all labeled cells was compared with that of non-labeled counterparts. MCF-7 cells labeled with anti-ERα antibodies demonstrated MFI of 187.77, while non-labeled MCF-7 cells had MFI of 878. PR expression in MCF-7 had the same pattern: cells labeled with anti-PR antibodies show significant increase in MFI, namely 10.55 vs. 158.0, compared with non-labeled MCF-7 cells (Fig. 1A,B). The mean fluorescence intensity of labeled BT-474 cells was significantly higher than of non-labeled counterparts (− 15.0 vs. 70.16) (Fig. 1C).

### Detection of circulating tumor cell molecular subtype

Wopereis et al. validated flow cytometry as the method, which can be used to distinguish molecular subtypes in primary lesion of breast cancer patients with sufficient sensitivity and specificity^14^. We compared molecular subtype of primary tumor with CTCs using developed approach with some modifications. Initially, it was necessary to define the boundary between negative and positive cells for each marker. Given that lymphocytes do not express ERα and only minor population of NK-cells and CD8+ lymphocytes expressed PR, we used lymphocyte gate in CD45-labeled cells as negative control for detection of ERα and PR expression in CTCs. Samples in which more than 10% of the CTCs had a stronger fluorescence intensity than lymphocytes were considered ERα- and PR-positive^15^.

**Figure 2:**
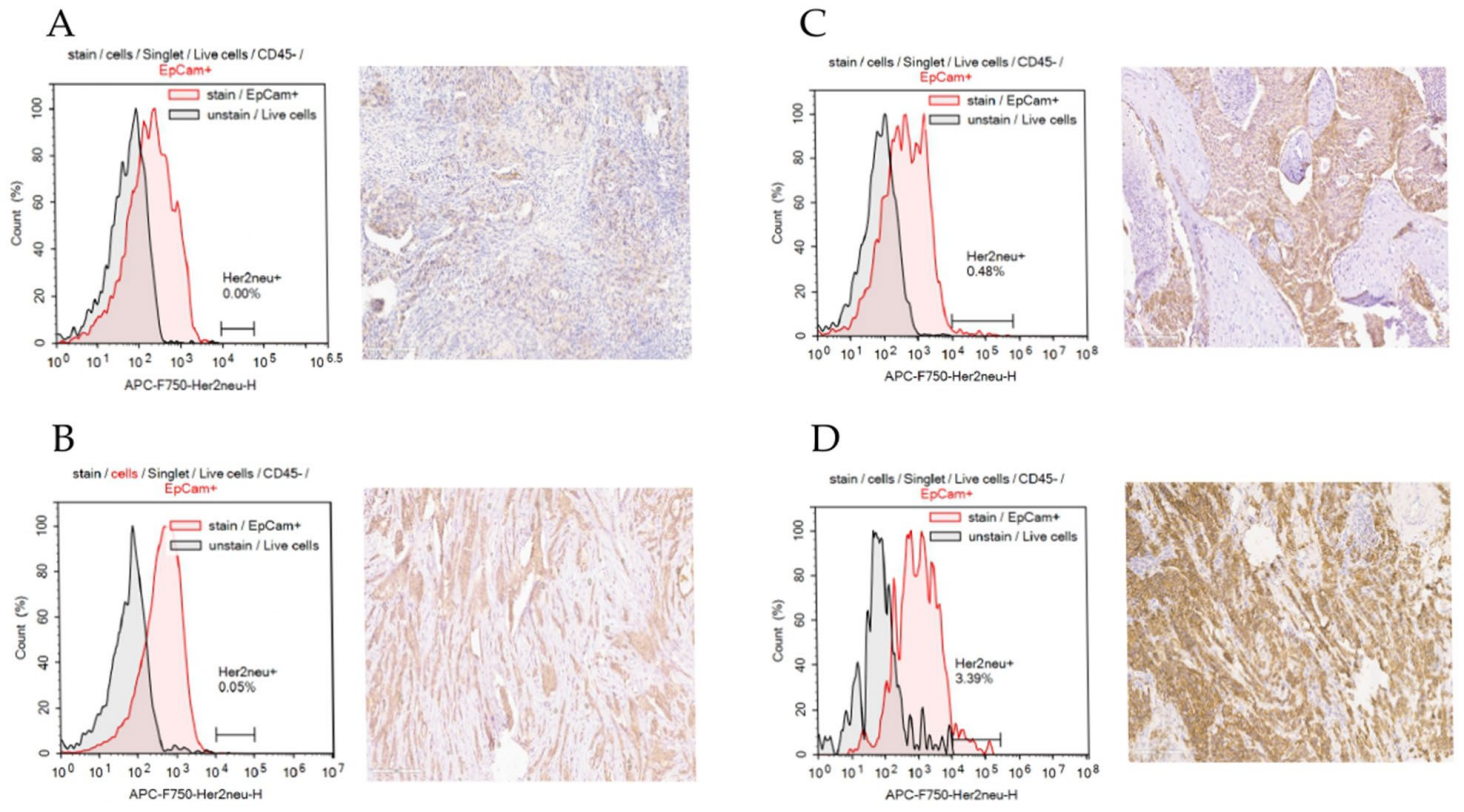
Comparison of HER2 expression detected by IHC and flow cytometry. (**A**) Corresponds to HER2-negative sample; (**B**) sample with HER2 expression corresponding to score 1; (**C**) sample with HER2 expression corresponding to score 2; (**D**) sample with HER2 expression corresponding to score 3.

Since determining HER2 status requires separating normal expression from overexpression, which is an indication for targeted therapy, it was necessary to establish a cut-off of fluorescence intensity. We selected surgical specimens in doublets with score 0, 1, 2, 3, and evaluated the MFI corresponding to each score by flow cytometry (Fig. 2). Samples with HER2 score 2 were tested by FISH and concludes as HER2-negative.

Sample where more than 0.5% of the CTCs had a fluorescence greater than 10,000 were considered HER2-positive. The proposed approach distinguishes samples with a score 2, which are considered HER2-negative, and those with a score 3, which are considered HER2-positive. While method described in Wopereis S. (2021) study used samples with score 0 as negative control thereby considered samples with score 1, 2 and 3 as positive.

CTC samples considered to be Ki-67+ if 20% of cells expressed the marker. Given the expression of ERα, PR, HER2 and Ki-67 in CTCs we defined the molecular subtype of the whole population of CTCs in each breast cancer patient.

### HER2 expression pattern in primary tumors in cases with conversion of molecular subtype to luminal B (HER2+)

**Figure 3:**
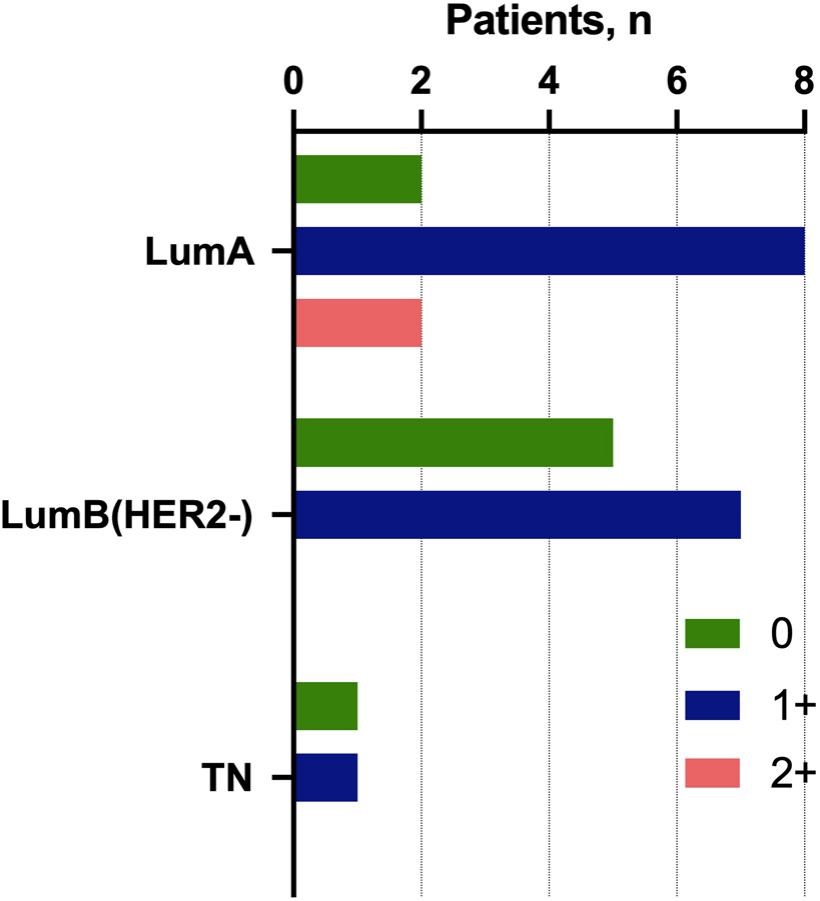
HER2 expression in primary tumor of breast cancer patients with conversion to luminal B (HER2+) subtype.

There were three variants of molecular subtype conversion resulted in luminal B (HER2+) subtype in CTCs: from luminal A, luminal B (HER2−) and triple-negative primary tumors. We evaluated the intensity of HER2 expression in the primary tumor to assess whether such phenomenon be explained by the heterogeneity of the primary tumor and, consequently, the appearance of a minor subpopulation of HER2+ CTCs in the bloodstream (Fig. 3).

The lack of HER2 expression in primary tumor cells in patients allows us to suggest, that CTCs may have acquired the ability to express HER2+ during intravasation. We suppose that at least in those cases where the conversion raised from a primary tumor with score 0, CTCs do not originate from a minor subpopulation of the HER2+ tumor cells. Moreover, triple-negative subtype which is characterized by the lack of the HR expression on tumor cells in primary lesion resulted in luminal B (HER2−) CTCs, which expressed at least one of the HR.

### Molecular subtypes of primary tumors and CTCs of breast cancer patients

**Figure 4:**
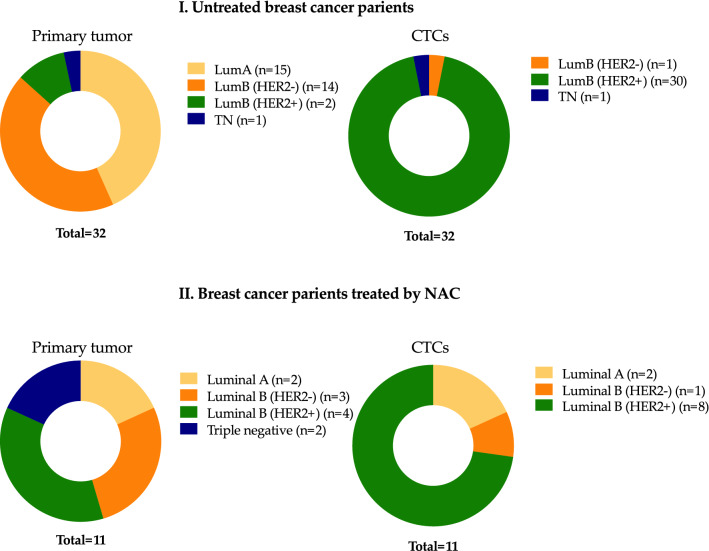
Molecular subtypes of primary tumors and CTCs in untreated (I) breast cancer patients and treated by NAC (II).

Most of the primary tumors in untreated patients were luminal A and luminal B (HER2−). The group of patients treated by NAC were evenly represented by all molecular subtypes (Fig. 4, panel I and II). Frequency of luminal B (HER2+) subtype was higher in group of patients treated by NAC, 2/32 and 4/11, correspondingly (p = 0.0256). CTCs in untreated patients were represented mostly by luminal B (HER2+) subtype (Fig. 4, panel I and II). The existing pattern is obvious, since the molecular type is an indication for prescribing therapy.

**Figure 5:**
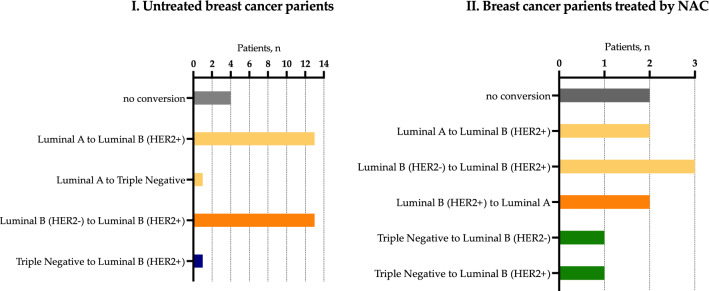
Variants of molecular subtype conversion in CTCs in untreated (I) breast cancer patients and treated by NAC (II).

Conversion of molecular subtype in CTCs was observed in 90% (29/32) of untreated patients; in patients treated by NAC conversion of molecular subtype occurs in 82% (9/11) cases (Fig. 5, panel I and II). There were no cases with HER2+ tumors, while conversion resulted in HER2+ CTCs was observed in one case after NAC.

Four different conversions out of the sixteen possible variants were observed in untreated breast cancer patients, in patients treated by NAC there were five different conversions (Fig. 5, panel I and II).

Molecular subtype conversion resulted in unfavorable variant in CTCs was detected in 97% (28/29) of untreated patients and in 56% (5/9) of patients treated by NAC. Interestingly, luminal A tumors converted in all possible molecular subtypes except HER2+. It turned out that all molecular subtypes of primary tumors could undergo the conversion (Fig. 5).

**Figure 6:**
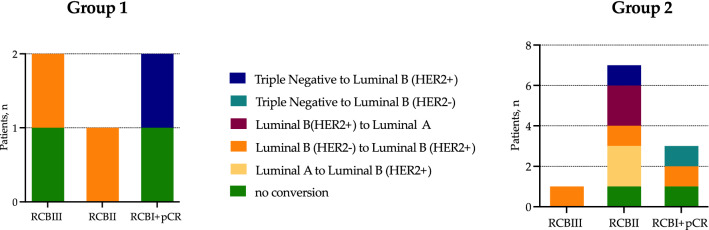
Variants of molecular subtype conversions in breast cancer patients from group 1 and 2 with different NAC responses.

**Figure 7:**
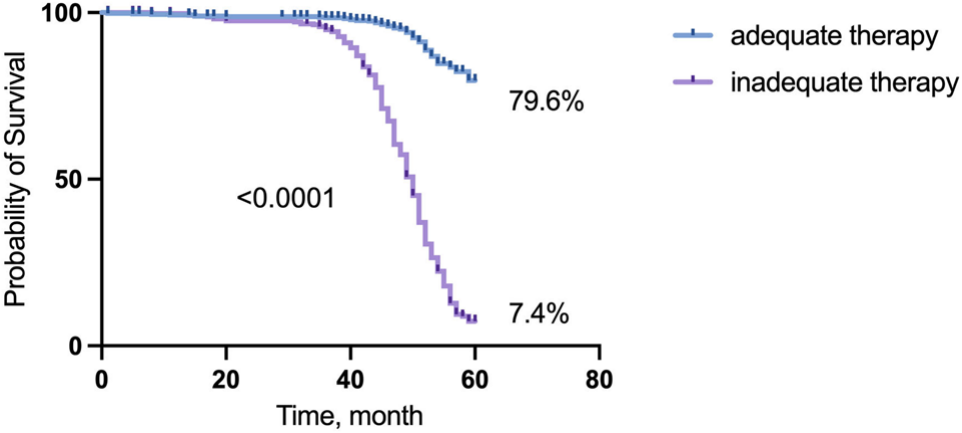
Metastatic-free survival of breast cancer patients treated according to molecular subtype of the primary tumor.

Interestingly, in untreated patients molecular subtype conversion resulted in luminal B (HER2+) in 89.7% (26/29) of cases, while in patients treated by NAC only in 66.7% (6/9) of cases. The different variants of conversions in patients with NAC occurred with equal frequency. While almost all the conversion in untreated patients (26/29) was represented with approximately equal frequency from luminal A and luminal B (HER2−) subtypes to luminal B (HER2+). Conversions from luminal B (HER2+) were detected only in group of patients treated by NAC, namely in 2/9 and 0/29 cases, correspondingly (p = 0.0028) (Figs. 6, 7).

Luminal B (HER2-) primary tumors either gave rise of CTCs only with luminal B (HER2+) subtype or does not underwent conversion regardless of neoadjuvant chemotherapy. Among the 5 cases that did not undergo conversion, there were two cases of luminal B (HER2−) and three cases of luminal B (HER2+). All luminal A and triple-negative cases have undergone conversion in both groups of patients.

### Frequency of conversions depending on clinicopathological characteristics in breast cancer patients

We compared the frequency of molecular subtype conversions in CTCs of untreated and treated by NAC breast cancer patients considering such clinicopathological parameters as tumor size (T), lymph node involvement (N), tumor grade and occurrence of hematogenous metastasis in follow-up period.

Probably, due to the small number of cases, no significant differences in groups of patients were found. However, the one of the most common subtype conversions from luminal A to luminal B (HER2+) were absent in patients with a large tumor size (Supplementary Figs. S1, S2). Cases without conversion occurs more often in patients with T2 both in untreated patients and in patients treated by NAC.

While analyzing conversion frequency in patients without lymph node involvement and node-positive patients (N1-3) it turned out that rare variants were more common for patients with lymph node metastases independently of NAC treatment (Supplementary Figs. S1, S2). Namely, 3 out of the 4 rarest conversions observed in patients with N1-3. In triple-negative cases with lymph node metastases there was a conversion to luminal B (HER2+) subtype, while in cases without lymph nodes involvement—to luminal B (HER2−).

Over the entire 24-month follow-up period, distant metastases occurred in 7% of breast cancer patients (4/43). It turned out that three cases with metastasis were represented by two conversion variants of molecular subtype in CTCs: one of the most frequent variants—luminal A to luminal B (HER2+) and one of the rarest—luminal B (HER2+) to HER2+ subtype. The last conversion variant occurred in patient treated by NAC. Both conversions resulted in unfavorable variants, while in two cases with conversion from luminal A to luminal B (HER2+) the administered chemotherapy was probably insufficient due to hormone therapy alone prescribed according to the luminal A primary tumor.

### Response to NAC depending on molecular subtype conversion in breast cancer patients

Evaluation of the NAC efficacy allows us to discuss the adequacy of the administered therapy according not only to the primary tumor, but also to the molecular subtype in CTCs. In our study 16 out of the 43 breast cancer patients were treated by NAC, in 5 patients CTCs phenotyping was performed before NAC treatment (group 1), while in 11 patients after treatment (group 2). Partial and complete tumor regression in untreated patients were observed in one and two cases, correspondingly; two patients demonstrated tumor stabilization (Fig. 6).

Good response to the NAC in group 1 observed both in case without conversion and case of triple-negative molecular subtype to luminal B (HER2+) conversion (Fig. 6). In group 2, good responses were detected in patients without conversion and in case of favorable triple-negative to luminal B (HER2−) subtype conversion (Fig. 6). Only one case with good response was represented by unfavorable conversation from luminal B (HER2−) to luminal B (HER2+).

### Association of treatment adequacy with short- and long-term breast cancer outcomes

To assess adequacy of treatment we used an approach suggested by Gomez-Acebo et al.^16^. The authors classified breast cancer patients after treatment into three groups according to the adherence to St Gallen-2013 recommendations with aim to assess overall survival. Namely, authors presented further groups: “In St Gallen”—if treatment consisted in exactly the main St Gallen recommendation for that patient; “Over St Gallen”—if the woman received the main St Gallen recommendation for her tumor subtype plus some additional therapy and “Under St Gallen”—if the woman did not receive the main St Gallen recommendation for her tumor subtype. We have combined two groups named “In St Gallen” and “Over St Gallen” into one group considered that these two variants of therapy will be effective against CTCs with conversed molecular subtype and considered as “adequate therapy”. We considered the therapy, which administered according to the primary tumor subtype and being insufficient against CTCs which underwent unfavorable conversion as “inadequate”.

Further, we used Pearson’s Chi-square test to determine statistically significant differences in the proportion of patients with different responses to the NAC according to the therapy adequacy (p < 0.0001). Thus, in patients with complete tumor regression the administered therapy was adequate in almost all cases (4/5), while in patients with stabilization of tumor growth the therapy was inadequate in 2 out of 3 cases. Therapy administered according to the primary tumor subtype in patients with partial regression was adequate in half of the cases (4/8) (Fig. 6).

Considering that prevailed cases were represented by early breast cancer patients, which have extremely low distant metastasis incidence^17^, we used tool for simulation censored survival-time distribution based on our own samples^18^.

So, we evaluated the 5-year metastasis-free survival rate in breast cancer patients, taking into account the adequacy of the indicated therapy. When therapy administered according to molecular subtype of primary tumor was also adequate against the converted subtype of CTCs, the 5-year metastatic-free survival was 79.6% (Fig. 7).

By contrast, in patients with inadequate therapy the survival rate was significantly lower and amounted 7.4% (p < 0.001).

Based on the univariate and multivariate analysis, we found that adequacy of indicated therapy showed significant prognostic power, even after adjusting for classical prognostic factors (HR (95% Cl) 9.702 (7.767–12.12), p < 0.0001).

## Discussion

Alterations of the cancer cells molecular interface during tumor growth and progression could be associated with many negative effects, for example, drug resistance, local recurrence, and metastatic spread. HR/HER2 status as well as Ki-67 expression in tumor cells are determinant for molecular subtype classification and, consequently, for treatment decision in breast cancer patients. There is a strict conventional algorithm for IHC evaluation of ERα, PR and HER2 expression. According to ASCO guidelines, tumors were considered positive for ERα and PR when at least 1% of the tumor cells showed unequivocal nuclear staining^19^. HER2 overexpression is scored according to the pattern of membranous staining and percentage of stained tumor cells as follows: 0, no staining or faint incomplete staining in < 10% of cells; 1, faint incomplete staining in > 10% of cells; 2, weak to moderate complete staining in > 10% of cells; and 3, strong complete staining in > 10% of cells. While score 2, confirmed by FISH analysis, and score 3 were considered positive. Such approach is widely used for molecular subtype detection in residual tumor, lymph node metastasis and distant metastasis^20,21^. Wherein many authors showed a significant discordance between primary tumor and metastatic lesion, reporting HR discordance rates between 30 and 40% and HER2 discordance rates between 10 and 30%^2,21,22^.

The etiology of primary and metastatic discordance can be attributed to intrinsic tumor heterogeneity, as well as alterations in the molecular interface of cells during tumor progression. Our result demonstrated the probability of the second claim by evaluating HER2 and ER/PR expression in triple-negative primary tumors in patients whose molecular subtype in CTCs has converted to luminal B (HER2+) (Fig. 3). Furthermore, both triple-negative cases converted to luminal B (HER2+) subtype in CTCs also lacked ERα and PR expression in the primary tumor.

Wopereis et al. validated flow cytometry as the method, which can be used to distinguish molecular subtypes in primary lesion of breast cancer patients with sufficient sensitivity and specificity^15^. We have shown that such approach can also be applied for molecular subtyping of the total pool of CTCs. According to the method developed by Wopereis et al., we used lymphocytes as negative control for ERα and PR expression while detecting CTCs HR status. We considered CTCs sample positive by each marker if at least 10% of cells were positive of the population, i.e., had a stronger fluorescence intensity than lymphocytes. We established the cut-off for fluorescence intensity corresponding to HER2 overexpression in tumor cells to define HER2 status. We compared expression intensity and percent of HER2-positive cells in breast cancer tumor samples detecting by IHC and flow cytometry. We performed some modification of method suggested by Wopereis et al. as follow. We selected samples with HER2 expression classified as score 0, 1, 2, 3 by IHC and performed the flow cytometry analysis using anti-HER2 antibody (Fig. 2). Thus, to define HER2+ CTCs samples we established the cut-off for fluorescence intensity and percentage of positive cells corresponding to tumor sample with score 3 and allowing to distinguish tumor samples with score 2 (HER2− by FISH) and score 3. Whereas approach suggested by Wopereis et al., considered samples with score 1, 2 and 3 as HER2-positive. Finally, all CTCs samples were classified to 5 molecular subtypes according to HR/HER2 status and Ki-67 expression: luminal A, luminal B(HER−), luminal B(HER+), triple-negative and HER2+ (or HER2−enriched).

It turned out that the frequency of molecular subtype conversion in CTCs compared to the primary tumor was extremely high and amounted 82–90%. At the same time, we noticed the pattern of the most frequent conversions to the unfavorable variant. Only in three cases, a conversion to the favorable variants was found.

The choice of breast cancer treatment strategy is based on the molecular subtype of the primary tumor, and when an unfavorable conversion occurs, this fact is not taken into account due to the objective reasons. We used the approach suggested by Gomez-Acebo et al.^16^ to assess the adequacy of administered chemotherapy. The scheme, which was sufficient against converted molecular subtype in CTCs, was considered as “adequate therapy”. If the unfavorable conversion variant takes place and the scheme of chemotherapy was insufficient, we supposed that therapy was “inadequate”.

The studied cohort was represented by an early breast cancer, which is characterized by a relatively low risk of distant metastasis. Taking into account mentioned above, in accordance with recommendations on the required sample size for survival analysis^23^, we used mathematical tools to increase the sample size. Indeed, simulated 5-year metastasis-free survival was decreased in patients with inadequate therapy and can be explained by spread of tumor cells across the body.

The correctness of such classification is also confirmed by the response of NAC depending on molecular subtype conversion. Mostly, patients with complete tumor regression were represented by cases without conversion of molecular subtype or with conversion to more favorable variant in CTCs. Consequently, the therapy in these cases was adequate. Patients with stabilization and partial regression were represented by great diversity of conversions. At the same time, cases with tumor stabilization mostly characterized by unfavorable variants of conversions and, consequently, characterized by inadequate therapy.

Probably, conversion of molecular subtype in CTCs, firstly, could be explained by tumor heterogeneity and selection of cells with distinct phenotypes. Secondly, the presence of molecular subtype conversion in CTCs could be an indicator of tumor cells plasticity realized on different levels, namely in primary tumor and CTCs. Furthermore, we suppose that the presence of molecular subtype conversion could be the factor supported secondary drug resistance. However, this assumption requires further careful study.

The question remains unknown, how can we extrapolate obtained results to the cases where no CTCs were detected. It is known that most CTCs are cleared from circulation within 1–2 h^24^. Probably in such cases, it is necessary to determine the presence of CTC repeatedly. An alternative approach is to establish high-precision correlations between molecular subtype conversion and the characteristics of the primary tumor. This will allow evaluating the probability and direction of conversion based on the parameters of the primary tumor. In this case, it makes sense to specify the molecular subtype of CTCs with some frequency, for example, between cycles of chemotherapy, since NAC as well as adjuvant chemotherapy, induces a wide variety of molecular subtype conversions. Moreover, we suppose that assessment of alteration of molecular interface on CTCs compared to primary tumor should be used for any other carcinomas if the point of therapy is a certain molecular target.

## Conclusions

The main conclusion of our study is that not only alteration of the individual receptors’ expression in CTCs is important, but also detection of conversion of the molecular subtype in CTCs compared to primary tumor. Our study revealed that good response to neoadjuvant chemotherapy observed in case of adequate therapy, namely, when chemotherapy scheme was sufficient against CTCs. This claim confirmed by significantly increased 5-year simulated metastasis-free survival in patients with sufficient therapy compared to patients with inadequate therapy.

## Supplementary Information

Supplementary Figures.

## Data Availability

The datasets analysed during the current study available from the corresponding author on reasonable request.
