# Supplementary material for: Molecular subtype conversion in CTCs as indicator of treatment adequacy associated with metastasis-free survival in breast cancer
Source: Sci Rep. 2022 Dec 5;12:20949. doi: 10.1038/s41598-022-25609-0 (PMC9723174; doi:10.1038/s41598-022-25609-0)
Supplement: Supplementary file 1 — Supplementary Figures. [file 41598_2022_25609_MOESM1_ESM.docx]

SUPPLEMENTARY

**
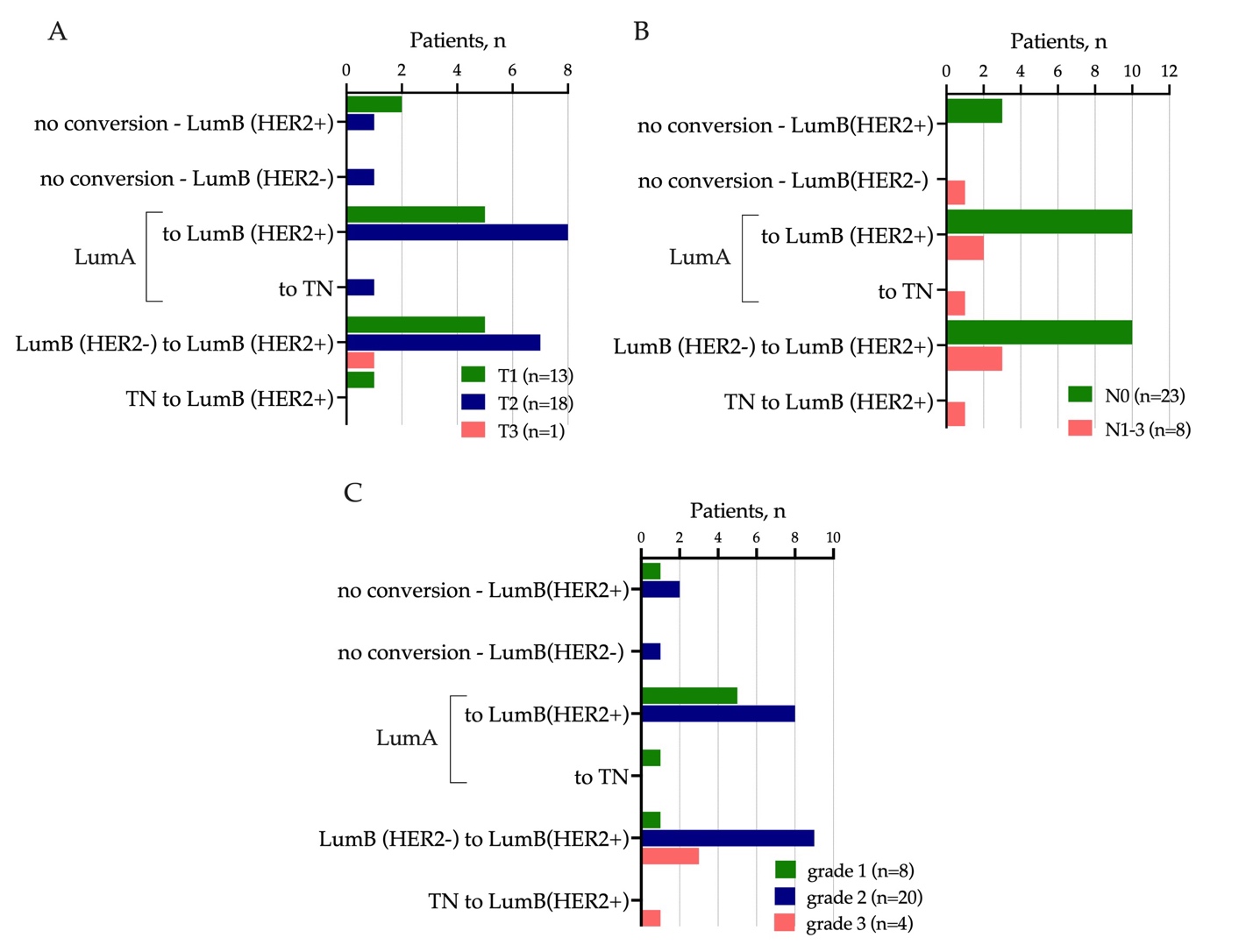
**

Figure 1. Frequency of molecular subtype conversions according to clinicopathological characteristics in untreated breast cancer patients. A - Variants of molecular subtype conversion in patients with different stages; B - Variants of molecular subtype conversion in breast cancer patients with different tumor grade; C - Variants of molecular subtype conversion in patients with different lymph node status

**
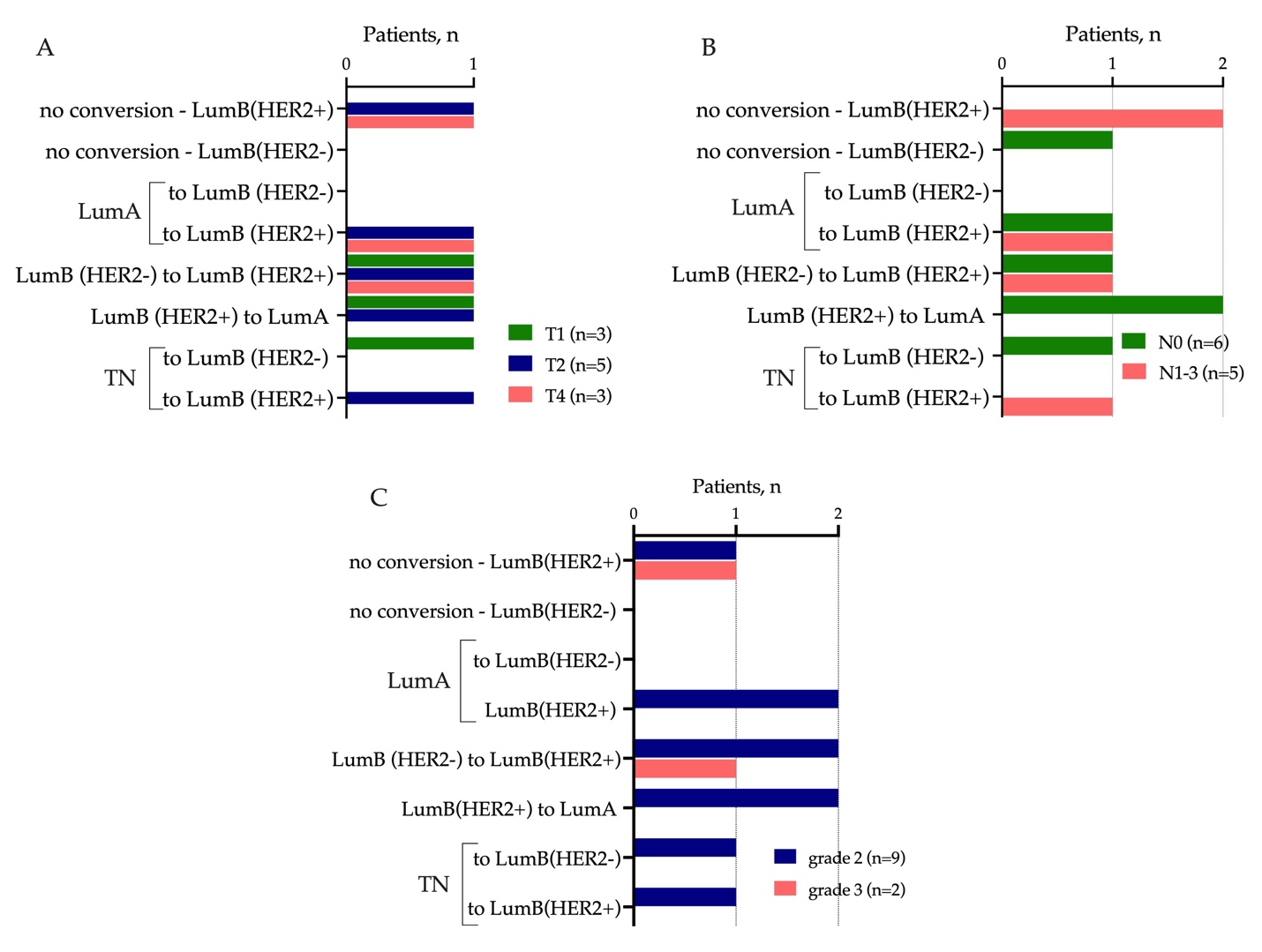
**

Figure 2. Frequency of molecular subtype conversions according to clinicopathological characteristics in breast cancer patients treated by NAC. A - Variants of molecular subtype conversion in patients with different stages; B - Variants of molecular subtype conversion in breast cancer patients with different tumor grade; C - Variants of molecular subtype conversion in patients with different lymph node status
